# Profilin desensitisation in patients with adverse reaction after plant-derived: our experience

**DOI:** 10.1186/2045-7022-5-S3-O20

**Published:** 2015-03-30

**Authors:** Simona Mezzacappa, Eleonora Nucera, Valentina Pecora, Angela Rizzi, Arianna Aruanno, Lucilla Pascolini, Manuela Ferraironi, Anna Giulia Ricci, Alessia Di Rienzo, Michele Centrone, Alessandro Buonomo, Domenico Schiavino

**Affiliations:** 1 Allergy Department, Università Cattolica del Sacro Cuore, A. Gemelli Hospital, Rome, Italy

## 

Profilins constitute a family of highly conserved proteins, which are present in all eukaryotic cells and are involved in processes related to cell motility. The first allergenic profilin was described in birch pollen and was designated Bet v 2.

Allergenic profilin were identified in tree and grass pollens, in weeds, in plant-derived foods, as well as in latex. Due to conserved structure of the profilins, specific IgE may cross-react with homologues from virtually every plant source. Therefore, profilin sensitization is a risk factor for allergic reactions to multiple pollen and food allergen sources.

Profilins are randomly distributed in pulp and peel and they are labile to heat denaturation and pepsin digestion. In fact the ingestion of vegetables in profilin sensitized patients usually determines reactions restricted to the oral cavity (oral allergy syndrome, OAS), despite in literature systemic reactions to zucchini and litches are reported.

We describe the history of six patients with adverse reactions after eating plant-derived food and positive allergological evaluation (skin tests, specific IgE, basophil activation test and double-blind placebo-control challenges (DBPCFC) for profilin, that have been undergone to desensitization treatment.

The protocol of desensitization started with a drop of profilin solution (50 µg/ml) diluted 1:10^18^ in water until the highest dose of 10 drops of undiluted solution three times a week. They underwent this desensitization treatment at home and were followed in Day Hospital regimen monthly. According to the protocol they were trained in medical treatment of allergic reactions and equipped with an emergency kit: autoinjectable epinephrine, betamethasone and clorphenamine. At the end of the treatment all patients had negative DBPCFCs with culprit foods and a decrease of specific IgE levels for profilin and vegetable foods. Moreover, the desensitization with profilin has proved to be safe as no serious adverse events were observed in our patients. Profilin desensitization allowed that our patients could manage their diet without restriction, eating several foods previously not tolerated.

